# Optical XOR logic gate design in two dimensional photonic crystal using ANN and PSO

**DOI:** 10.1038/s41598-025-12146-9

**Published:** 2025-07-21

**Authors:** Farsad Heidari, Delir Pashabadi, Mohammad Fathi, Mohammad Razaghi

**Affiliations:** Department of Electrical Engineering, University of Kurdistan, Sanandaj, Iran

**Keywords:** Photonic crystal, Optical logic gate, FDTD, ANN, PSO, Electrical and electronic engineering, Computational science, Computer science

## Abstract

In this paper, we present an all-optical XOR gate based on two-dimensional photonic crystals (PCs). The proposed gate is designed to be used in optical computing and all-optical logic applications, offering advantages such as fast computation and parallel processing. To enhance the design process and optimize its performance, we employ artificial neural network (ANN) to model the gate’s behavior. A dataset of 555 samples, obtained from the Finite-Difference Time-Domain (FDTD) method, is used to train the ANN model. Subsequently, particle swarm optimization (PSO) is used to find optimal values of XOR design parameters, including the lattice constant and the radius of the rods. This optimization process leads to an optimized structure that enhances the gate’s performance. The proposed all-optical XOR gate exhibits a range of output powers across the respective logical states (desired state): 0 (0), 0.995 (1), 0.935 (1), and 0.015 (0). The proposed structure achieved an acceptable contrast ratio of 17.947 dB. By leveraging the ANN-PSO approach to improve the performance of PCs, we demonstrate a promising strategy for developing complex and efficient optical computing systems.

## Introduction

Recent advances in all-optical systems focus on photonic crystals (PCs). PCs are tiny optical structures with a regularly changing refractive index^1–3^. Made from materials like dielectrics, PCs guide electromagnetic waves much like semiconductors control electrons through their repeating structure^4–6^. The refractive index in this structure varies periodically, and these variations can occur in one, two, or three dimensions. Two-dimensional (2D) PCs can be created by arranging alternating dielectric rods. Given their significance, numerous studies have been conducted in recent decades to explore the potential of 2D PCs. Due to their small size, PCs have the potential to serve as the foundation for PICs^7–9^. Logic gates are key components in computer systems and are traditionally built with CMOS transistors. However, as miniaturization nears its limits, future low-power, high-speed computing faces challenges. Consequently, photonic approaches, especially PCs, are gaining interest. PC-XOR gates are important in optical computing because they can perform fast and low-power logic tasks like binary addition, data comparison, and encryption. Their compact size and high speed make them ideal for use in photonic chips and advanced optical networks^10–13^.

In^14^, several all-optical logic gates with multiple capabilities based on 2D PCs are designed and simulated. The logic gates include AND, OR, XOR, NOR and NAND at a wavelength of 1550 nm. Their performance is based on optical wave interference and cavity resonance and is evaluated by considering the contrast ratio, transmission ratio and insertion loss. Multi-functionalities were achieved by tuning the refractive index or adjusting the input wave phase, making these designs promising for advanced photonic integrated devices. An all-optical Toffoli gate based on a 2D silicon–air PC, utilizing constructive and destructive light interference is presented in^15^. The gate integrates a two-input–three-output optical AND gate and a two-input–one-output optical XOR gate, with Y-junction power splitters at the AND gate inputs. With intensity encoding and no nonlinear materials, the proposed Toffoli gate offers improved performance, including optimized response time and contrast ratios.

In^16^, a quantum CNOT gate using 2D PCs and an XOR logic gate is introduced. The structure, which consists of silicon rods, has a size of 25 × 35 μm and a fast response time of 0.53 picoseconds, which is suitable for high-speed applications. The clear separation between the input and output ports is a priority of this structure and claims to solve the key challenges in implementing quantum gates for quantum computing. In^17^, an all-optical AND gate based on a hybrid nonlinear PCs ring resonator structure is proposed. Because of the compact area of ​​175.45 µm2, contrast ratio of 13.17 dB, and response time of 2 picoseconds, this structure is suitable for integrated optoplasmonic applications. A compact 2D PCs design for all-optical logic gates using a combination of linear defects and a ring resonator is presented in^18^.

In photonic integrated circuits (PICs), PCs logic gates can be used as basic components to implement advanced computing operations. Due to their high integration capability, these circuits enable the design of all-optical processing systems in very small dimensions. In fact, just as logic gates are used in electronics to build complex units, these gates play a similar role in photonics. If multiple XOR gates are needed in a system, beam splitters and optical waveguides can be used to distribute the incoming light to the gates. In^19^, a photonic crystal-based XOR gate is presented, which is designed as a cascade and can be combined with other gates in a PIC. The authors have enabled the development of larger logic systems in an optical substrate by using crystal waveguides and resonators. High transmission rate and significant output contrast ratio are key features of this design. Also, in^20^, a review is given on the application vertical cavity semiconductor laser (VCSELs) in the field of optical integration. The use of VCSELs in optical integrated structures can be used as a light source for optical logic gates such as XOR. Many recent designs, such as the logic gate structure in^21,22^, have utilized interference-based defects and nanostructured resonators to implement optical logic by creating controlled optical paths. In particular^21^ achieved a high contrast of 62.38 dB for an XOR gate using T- and X-shaped waveguides and nano-resonators. In^22^, the coupling mechanism is based on the combination of optical waves in both clockwise and counterclockwise directions inside the ring resonators, which leads to constructive or destructive interference. This phenomenon, with the presence of nano-resonators, allows for control of the output intensity and precise operation of logic gates, including XOR, without the need to use nonlinear effects such as Kerr.

Nowadays, artificial neural networks (ANN) are widely used in the design and optimization of electronic circuits and will create suitable innovative opportunities for the creation of compact optical circuits. By using ANNs and related optimization tools, the performance of circuits will be increased with higher accuracy and speed, leading to its improvement. One of the applications of ANNs in this field is the recognition of complex patterns for the design of optical modules, so that it is possible to design and manufacture more efficient circuits with lower energy consumption^23–25^. On the other hand, in^26^ the PC structure was optimally designed and coupling mechanism is defect-based linear coupling, which is achieved by creating defects in the regular array of rods.

In optical systems such as PC structures, many gates can simultaneously process independent input light because light, unlike electric current, can travel in separate paths without interference. If multiple independent optical inputs are injected into separate (or parallel) structures, the outputs are calculated simultaneously. Therefore, designing multiple similar gates on an optical chip allows for parallel processing. The light can be guided to different gates through optical waveguides. At the output, CMOS-compatible photodetectors are used that convert the light intensity into an electrical digital signal. The arrangement of these detectors can be in the form of a matrix at the output of the chip^27,28^.

The main objective of this research is to demonstrate a fast, practical, and easy-to-implement design method that combines ANN with particle swarm optimization (PSO), emphasizing a novel hybrid AI-photonics approach. Our proposed approach is not simply to present a new numerical method for solving Maxwell’s equations, but rather the main focus is on modeling the structure by ANN and optimizing the structure design parameters by PSO. This combination results in improving performance compared to structures designed solely based on the direct solution of Maxwell’s equations such as FDTD.

This paper introduces a novel and optimized method for designing an all-optical compact XOR logic gate using ANN modeling technique. Specifically, it uses the power of ANNs to model the behavior of a PC logic gate to simulate the desired structure as simply and as accurately as possible. After that PSO, as a random search optimization algorithm, is presented to optimize the parameters crucial for XOR gate design. We present a concise and effective design for an all-optical XOR gate that functions based on power values and delivers precise logic outputs. In our structure, the light transmission value is considered as the basis for distinguishing between logical zero and one, so that high light transmission is equivalent to logic 1 and low or almost zero transmission is equivalent to logic 0. This method is common in many optical structures based on PCs. The proposed all-optical XOR gate produces output powers corresponding to the logical states: 0, 0.995, 0.935, and 0.015. The results of this research demonstrate the successful realization of an all-optical XOR gate using the proposed PC structure and the assistance of the suggested ANN-PSO modeling and optimization framework.

The different sections of this research are as follows: Sect. 1, includes an introduction to PCs and their role in the development of optical logic gates. Section 2, describes the materials and methods used to analyze PCs. Section 3, the design of an all-optical XOR logic gate using PC structures is fully described. In Sect. 4, the performance of this optical logic gate is modeled and implemented using an ANN to enable the network to understand the gate’s behavior and predict its output. Section 5 presents a PSO algorithm to enhance the modeling process, optimizing the structure’s parameters for improved outputs of the optical logic gate. Section 6 refers to the results review and discussion of the proposed structure after considering PSO. Finally, in Sect. 7, a general conclusion is drawn about the research in question.

## Materials and methods

To study the propagation of electromagnetic waves in a 2D periodic medium, Maxwell’s equations must be solved under the assumption of an isotropic, non-magnetic, and charge-free medium. Consequently, the constitutive equations can be expressed as follows^4,29^:

1 $$\:D\left(x,\:z,\:t\right)={\epsilon\:}_{0}{\epsilon\:}_{r}\left(x,z\right)E(x,z,t)$$

2 $$\:B\left(x,\:z,\:t\right)={\mu\:}_{0}H(x,z,t)$$

The permeability $\:{\epsilon\:}_{0}$ and permittivity $\:{\mu\:}_{0}\:$of free space are denoted, while $\:{\epsilon\:}_{r}$ represents the relative permittivity of the material. The following equations are representing waves propagating through a periodic medium^4,29^:

3 $$\:\nabla\:\times\:E\left(x,z,t\right)=\frac{\partial\:}{\partial\:t}B(x,z,\:t)$$

$\:\nabla\:\times\:H\left(x,z,t\right)=\frac{\partial\:}{\partial\:t}D(x,z,\:t)$(4) The FDTD method can be used to determine the electromagnetic fields inside the PC. This approach involves using FDTD updating equations to calculate the electromagnetic fields at each moment in time, based on their current and previous values.

As the designed XOR gate operates on a 2D PC in the *xz* plane, there is no variation along the y-direction, $\:\partial\:/\partial\:y=0$. By substituting Eqs. (1) and (2) into Eqs. (3) and (4), two sets of field equations are obtained. The first set includes $\:{E}_{x}$​, $\:{E}_{z}$​, and $\:{H}_{y}$​ (TM mode equations), while the second set includes $\:{E}_{y}$​, $\:{H}_{x}$, and $\:{H}_{z}$​ (TE mode equations), assuming wave propagation along the *x*-direction. Given that the designed PC exhibit a wide bandgap in the TE mode, the corresponding equations are as follows:

5 $$\:\frac{\partial\:}{{\partial\:}_{t}}{E}_{y}=\frac{1}{{\partial\:}_{y}}(\frac{\partial\:}{{\partial\:}_{z}}{H}_{z}-\frac{\partial\:}{{\partial\:}_{z}}{H}_{z})$$

6 $$\:\frac{\partial\:}{{\partial\:}_{t}}{H}_{x}=\frac{1}{{\mu\:}_{x}}\frac{\partial\:}{{\partial\:}_{z}}{E}_{y}$$

7 $$\:\frac{\partial\:}{{\partial\:}_{t}}{H}_{z}=\frac{1}{{\mu\:}_{z}}\frac{\partial\:}{{\partial\:}_{z}}{E}_{y}$$

In this case, $\:{\epsilon\:}_{z}$, $\:{\mu\:}_{x}$​, and $\:{\mu\:}_{z}$ are related to $\:{E}_{z}$​, $\:{H}_{x}$​, and $\:{H}_{z}$ via the constitutive relations (1) and (2).

## Designing XOR gate using PCs

**Fig. 1:**
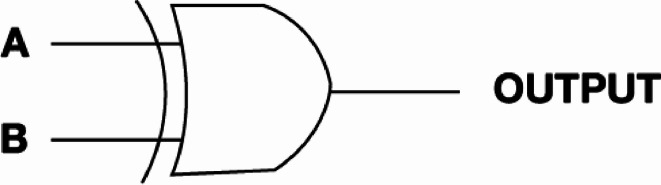
Circuit symbol XOR logic gate.

One of the basic digital gates is the XOR gate. A logic gate has a true output (1 or HIGH) when the number of correct inputs is odd. The output is true only when exactly one input is correct. Figure 1 shows the circuit symbol for the XOR gate, while Table 1 gives the corresponding truth table.

**Table 1:** The truth table of XOR logic gate.

INPUT		OUTPUT
A	B	
0	0	0
0	1	1
1	0	1
1	1	0

**Fig. 2:**
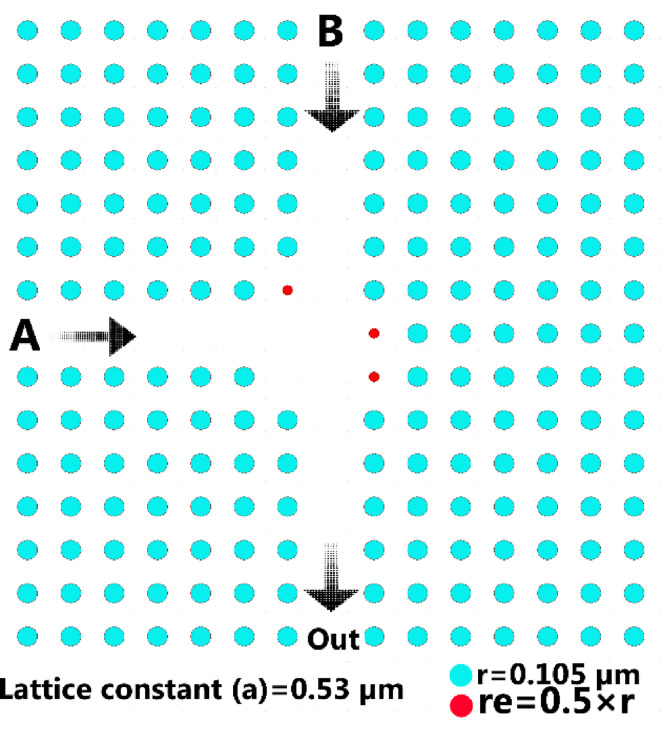
The proposed structure of XOR gate.

The illustrated starting arrangement in Fig. 2 includes the inputs *A* and *B*, along with an output designated as “OUT”. The depicted layout represents a PCs with a grid arrangement of dielectric rods in a 15 × 15 formation. In the illustrated structure in Fig. 2, the lattice constant is denoted as *a* varying from 0.500 to 0.5400 μm, and *r* represents the radius of the rods, varying from 0.090 to 0.0.122 μm. These ranges are deliberately chosen so that the structure has an effective bandgap in the telecommunication wavelength range (specifically 1.55 μm). Given that the *a*/λ ratio is the main criterion in PC design, these values ​​allow the *a*/λ ratio to be tuned in the optimal ranges for creating and exploiting the bandgap. The significant refractive index difference between GaAs (3.4 at 1.55 μm) and air (*n* = 1) leads to a desired photonic bandgap (PBG) in the PC structure within the telecommunication wavelength range. This bandgap plays an important role in confining and guiding light through defects in the rod array. Using GaAs rods in air, it is possible to guide light at a wavelength of 1.55 μm through a deleted row in the array.

Also, other studies have shown that different arrangements of these rods (such as hexagonal or square structures) can create suitable and tunable bandgaps. These properties have been analyzed by methods such as PWE to obtain PBG and FDTD to calculate the transmission^30^.

In this structure, GaAs material properties are more suitable for PC design and improving optical logic gate performance^29–31^. This choice is also inspired by some previous studies. Since the refractive index of GaAs at 1.55 μm is about 3.4, it has strong light confinement and provides a wider band gap in air-dielectric structures. Due to the availability of CMOS-based features, silicon and germanium are widely used, however, in this research, the focus is on designing an optimized structure with high refractive index and utilizing ANN-PSO, for which GaAs is a suitable choice^31^.

Due to the consideration of perfectly matched layer (PML) in the FDTD simulation, a 15 × 15 array was chosen for the photonic crystal lattice of this structure. One of the advantages of PML in the simulation environment is the absorption of incident light waves at the edges of the computational domain without back reflection, and it also allows the design of more compact structures, which makes them both more efficient in terms of optical performance and more computationally efficient. One of the reasons for choosing a 15 × 15 array in this study is also based on the previous structure presented in^32^, where such a configuration has shown good performance in creating a stable and good photonic band gap.

In creating the suggested structure, efforts have been made to opt for a compact structure with uncomplicated imperfections. To achieve this goal, GaAs rods have been employed in cubic shape, forming a photonic band gap for TE modes when placed in an air background with refractive index if GaAs has a refractive index of approximately 3.4 at a wavelength of 1.55 μm, a wavelength commonly utilized in optical telecommunication networks. This property makes it well-suited for use in photonic and optoelectronic applications within this range^33^. This structure is designed for use within the 1.55 μm optical communication window.

Optical band gaps refer to specific frequency ranges within which electromagnetic wave propagation is restricted within a crystal. Consequently, PCs crafted from low-power-dissipation dielectric materials offer an effective means of controlling the propagation direction and frequency of electromagnetic waves. The band gap structure provides valuable insights into how electromagnetic waves behave within a periodic arrangement and underscores the connection between frequency and wave vector.

**Fig. 3:**
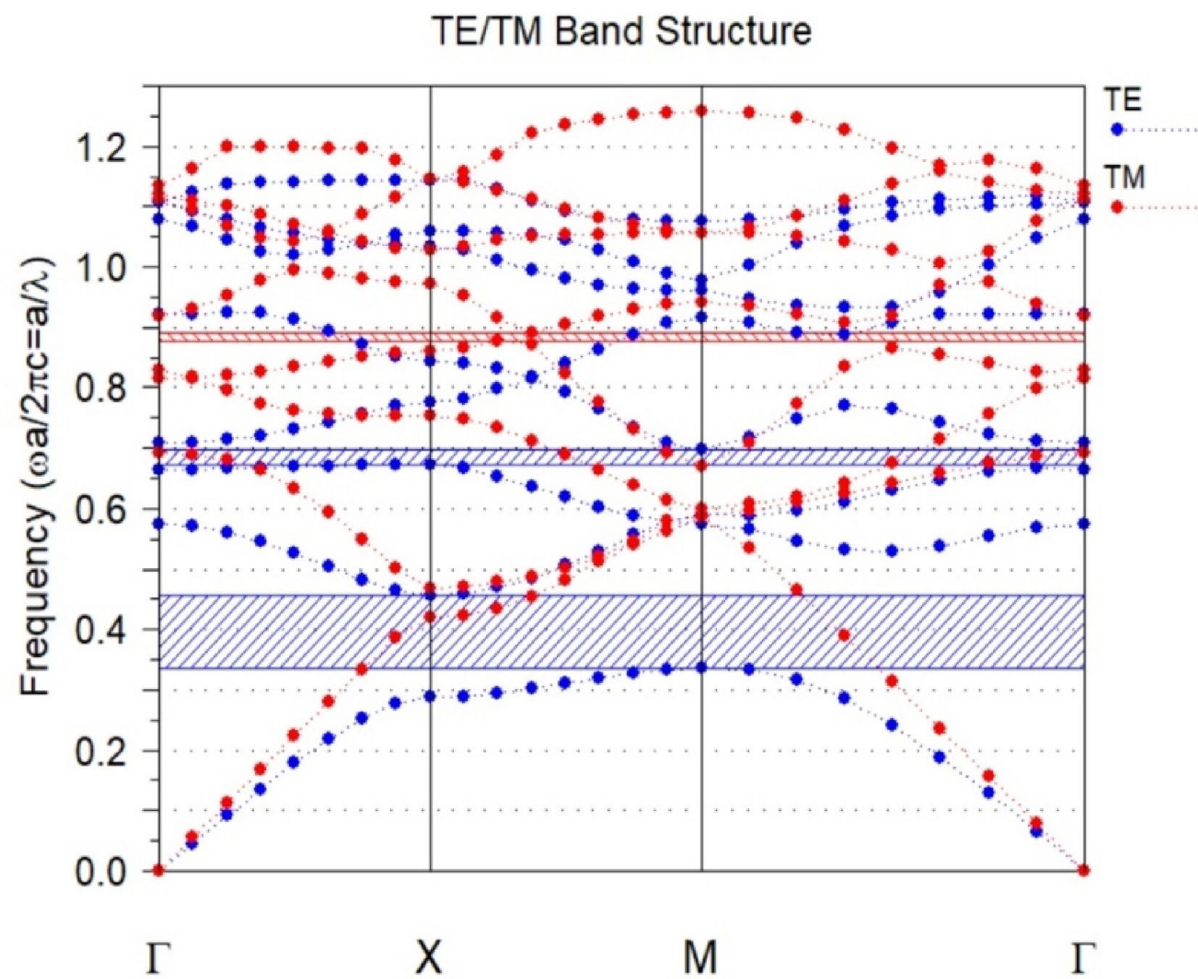
Photonics bandgap of the proposed structure.

Figure 3 illustrates the proposed configuration for bandgap photonics structure with *a = 0.53* and *r* = 0.105. The band structure diagram plays a crucial role in identifying the relevant wavelength range of $\:\lambda\:=1.38\:to\:\lambda\:=1.85\:\mu\:m$. In the early stages of design, it is essential to investigate a wider range of wavelengths to determine the location of the bandgap. Then, considering this range, geometric parameters such as *a* and *r* are chosen in such a way that the structure has high efficiency in the desired operating ranges, such as optical communication wavelengths. Optical communications ranges not only include specific wavelengths such as 1.3–1.55 μm, but also are divided into bands extending from the O-band (1260–1360 nm) to the U-band (1625–1675 nm). The chosen range of 1.38 to 1.85 μm intelligently covers a wide range of communications bands including E, S, C, L and even parts of the U-band, allowing for a comprehensive examination of the structure’s performance in these bands^34^.

The waveguides created by eliminating a row of dielectric rods is shown in Fig. 2. To achieve desired outputs, *r*_*e*_ is set to 0.5*r*^32^. The defect locations are determined based on the objective of creating constructive/destructive interference to match the XOR logic behavior. These locations are iteratively selected with the help of simulation to achieve correct logic output for all input states. The structure is designed to be symmetrical and compact to be usable for integration into optical circuits. Table 2, derived from the numerical simulation based on FDTD calculations, presents the outputs for the designed XOR gate.

In recent years, nanoscale structures have become achievable due to the advancement of nanofabrication technologies. In^35^, a structure of dielectric rods in air was designed and fabricated using precision electron beam lithography, which, using a chromium mask and precise patterning, can create structures below 50 nm, which are suitable for use in devices such as filters and optical sensors. Also, in^36^, a hexagonal photonic crystal structure consisting of dielectric rods in air was investigated. In this structure, the radius of the edge rods was adjusted to less than 50 nm and the light trapping performance in a wide bandwidth using boundary modes was investigated.

**Table 2:** Structure output for *a* = 0.53 and *r* = 0.105 μm.

A	B	Normalized Output	Logic value	CR (dB)
0	0	0	0	21.9
0	1	0.7750	1	
1	0	0.8320	1	
1	1	0.0050	0	

One parameter that should be considered is the interval between the values of logical “0” and “1”. Increasing this interval leads to a decrease in the identification error in the output. The comparison criteria for this interval are based on the CR, which is defined as follows:

8 $$\:CR\hspace{0.17em}=\hspace{0.17em}10log\frac{{P}_{1}}{{P}_{0}}$$

where *P*_*0*_ is the optical power for logic “0” and *P*_*1*_ is the optical power for logic “1”.

**Fig. 4:**
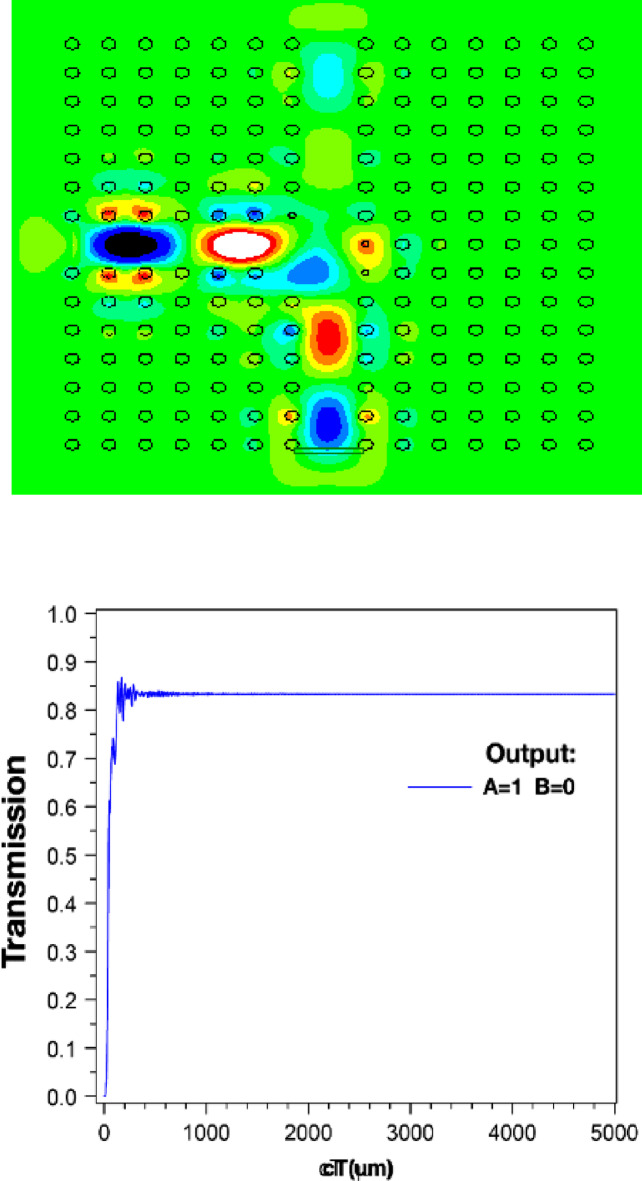
Magnetic field distribution and output of XOR structure at *A=1*,* B=0*, output=0.8320.

**Fig. 5:**
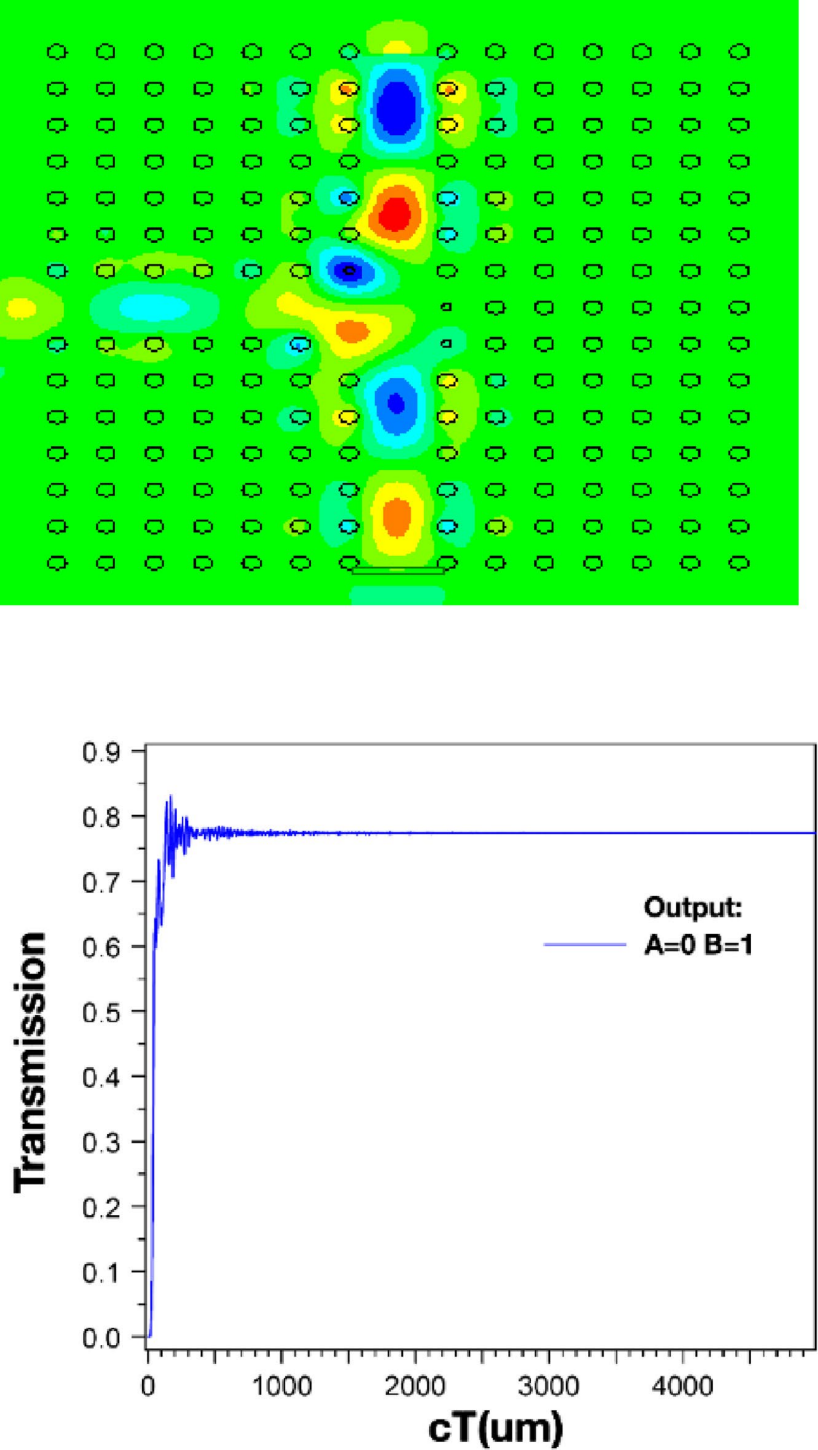
Magnetic field distribution and output of XOR structure *at A=0*,* B=1*, output=0.7750.

**Fig. 6:**
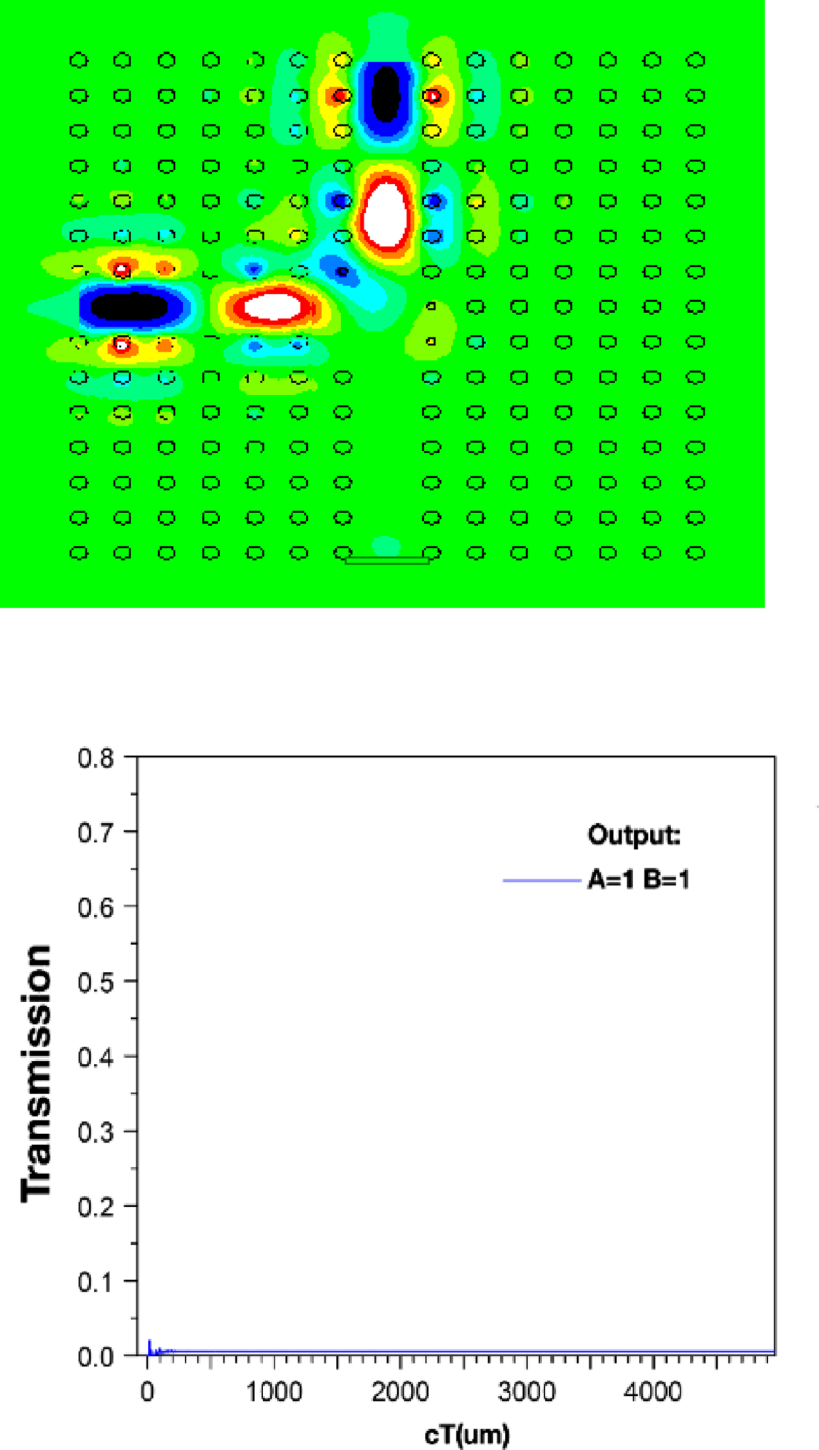
Magnetic field distribution and output of XOR structure at *A=1*,* B=1*, output=0.0050.

The structure’s output is displayed in Figs. 4 and 5, and 6. Additionally, the outputs corresponding to the *a* and *r* values listed in Table 2 are also shown. When port *A* is on and port *B* is off, the structure has an output as shown in Fig. 4. In this scenario, the output is expected to be close to one, and in this structure, it has a value of 0.840. However, when port *A* is off and port *B* is on (*A = 0*,* B = 1*), the output of the structure is as shown in Fig. 5 and has a value of 0.975. In this XOR gate, when both input ports are on (*A = B = 1*), the output of the structure should be zero. As shown in Fig. 6, the output of the structure shows a small light emission with a value close to 0 (approximately 0.005) when both inputs of this gate are on.

The parameter *“cT”* in Figs. 4, 5 and 6 is the product of *c* (speed of light) and *T* (total travelling time). Consequently, this parameter is an indicator of the effective path length covered by the signal or wave within the analyzed medium.

Considering the observations in Figs. 4, 5 and 6, the output value of the PC-based XOR gate depends on its structural characteristics, such as the *a* and *r*. These parameters can be considered as optimization variables to achieve the gate output values close to 1, and 0 in Figs. 4, to 6, respectively. To this end, in the following sections, we first model the PC-based XOR gate using a feedforward ANN. We use a limited dataset obtained from the design structure in Fig. 2 to train and test the target ANN. The dataset was generated through numerical calculations based on the FDTD method, which provides precise data generation and detailed analysis of the structural dynamics. Subsequently, we use the trained ANN model in the integration of PSO as a random search optimization algorithm to compute the optimal design parameters of the PC-based XOR gate, including the *a* and *r*.

In the presented structure, light amplification has not been considered as a part of the structure, because our focus is on the design and optimization of the basic XOR structure. However, to compensate for the light loss, solutions such as adding gain layers to amplify light in specific areas or increasing the power of the input light source within the allowable limit, depending on the operating power of the circuit, can be done^37,38^.

## ANN-based XOR gate modeling

ANNs are made up of interconnected neurons, making it easier to process complex data. Applications of this network include speech and image recognition, natural language, and pattern recognition, which derive from the ability to learn and generalize from large datasets^39^. Each neuron performs mathematical operations on each input and passes information through the layers to identify patterns and make decisions. ANN architectures vary from simple single-layer designs to more complex configurations. They also have multiple layers with multiple hidden layers, similar to deep neural networks. The presence of hidden layers between the input and output layers is essential for recognizing complex patterns in data. Training an ANN involves fine-tuning the weights of connections between neurons to mitigate errors and improve accuracy^40,41^.

### Dataset

In this structure, the gate was first modeled, but the goal in modeling the optical XOR gate is that the output has maximum optical power when the input *A = 0* and *B = 1* or vice versa and has a maximum value close to 1 and has minimum optical loss. This is while when both inputs are 1, that is (*A = B = 1*), the output should have the lowest possible optical power with minimum optical leakage. To train the target neural network, we first generate a dataset using the PWE-FDTD method. For each state of the gate inputs (*A* and *B*), we generate several samples with different values ​​of *a* and *r*. The inputs to the ANN are *a*,* r*,* A* and *B*. The output of the model is the prediction of the ANN to given inputs, indicated by a function as ($\:\text{n}\text{e}\text{t}\:(a,\:r,\:A,\:B)$).

**Fig. 7:**
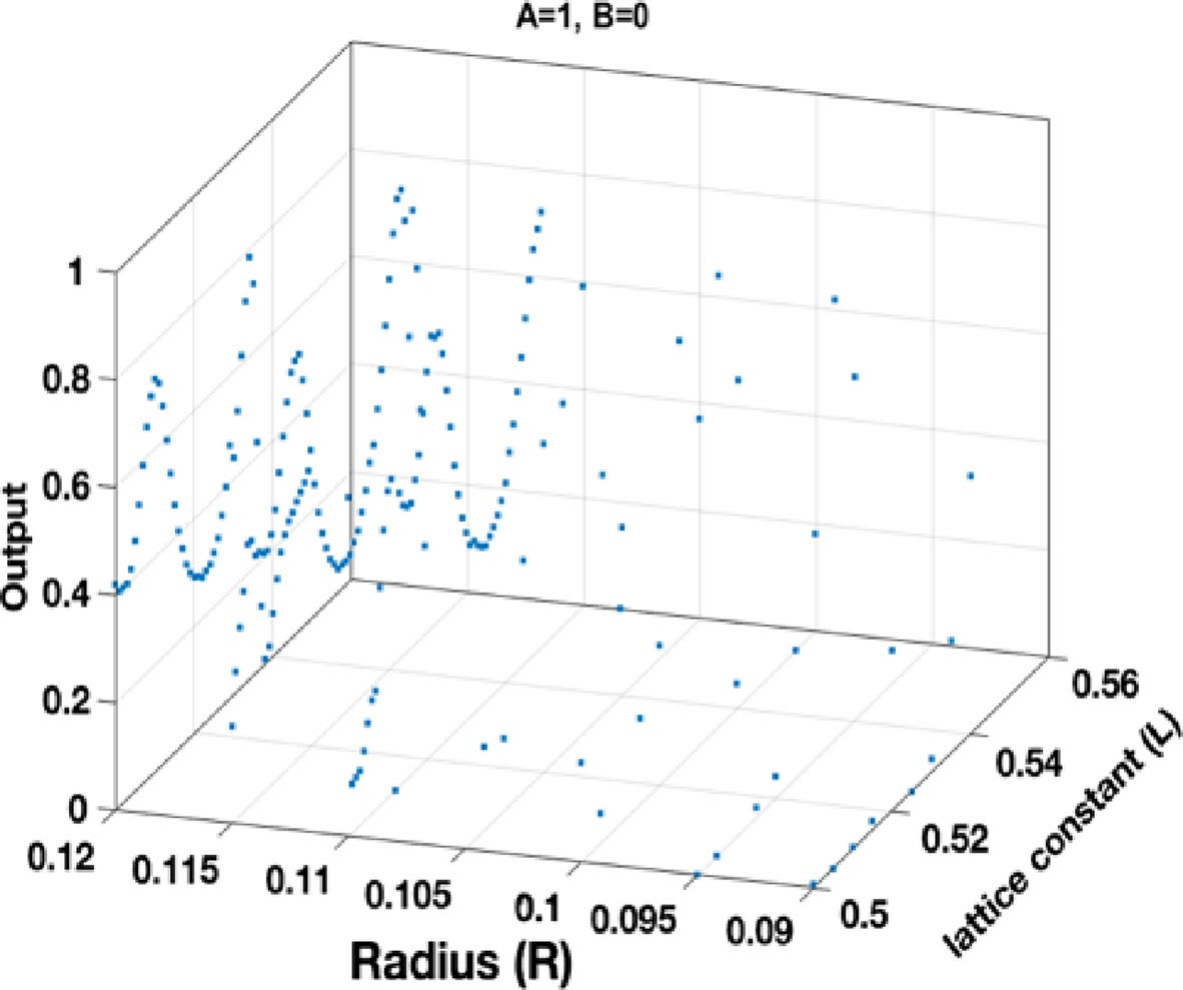
Structure output points at different *R* and *L* at *A=1*,* B=0*.

**Fig. 8:**
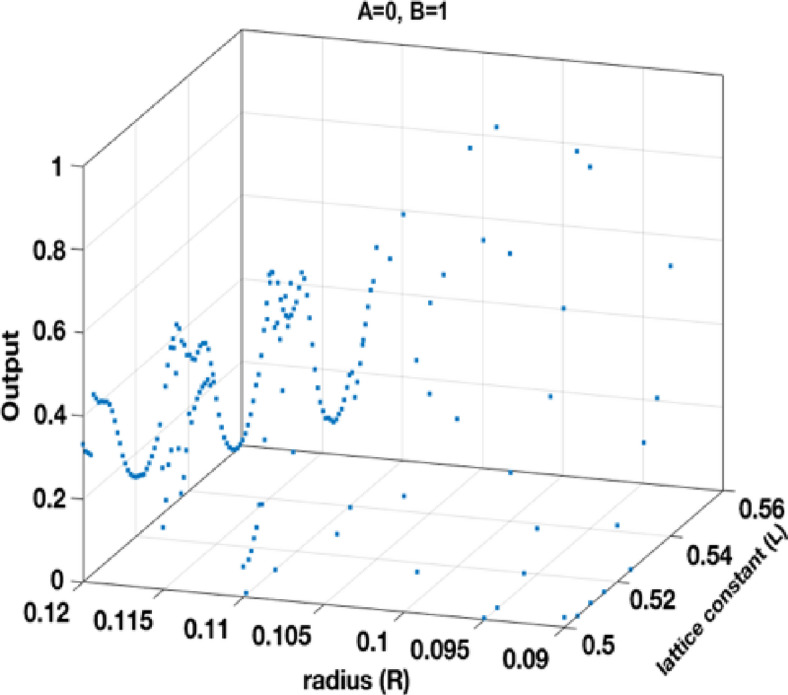
Structure output points at different *R* and *L* at A=0, B=1.

**Fig. 9:**
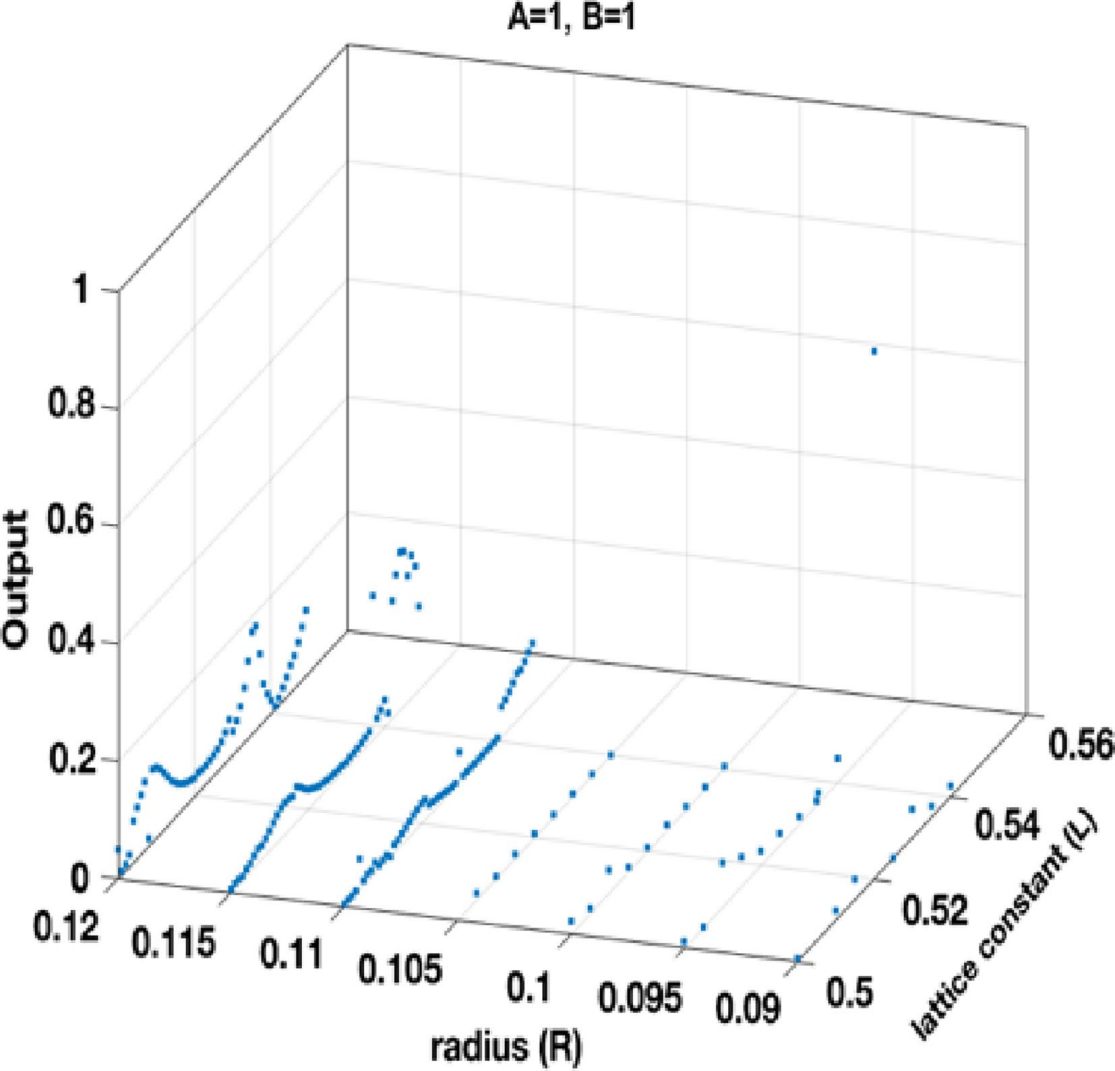
Structure output points at different *R* and *L* at *A=1*,* B=1*

The generated dataset consists of 555 samples, each of which contains four features and a single target variable. The features include *a*, *r*, input *A*, and input *B*. The target variable represents the output value of the gate. Figures 7 and 8, and 9 separately show the output of the dataset for the states *A = 1*,* B = 0; A = 0*,* B = 1;* and *A = 1*,* B = 1*, respectively.

### ANN structure

In this research, ANN is considered to model the performance and behavior of a PC-XOR gate. Our main goal is to analyze the performance of PC under different design parameters and accelerate the response time. Design and simulation of PC structures are inherently complex and time-consuming. With the development of this model, the response time has been reduced and the simulation optimization time has been reduced.

The considered ANN has two hidden layers, each layer containing 10 neurons. After receiving inputs from the previous layer and applying mathematical functions, these neurons transmit the outputs to the next layer.

**Fig. 10:**
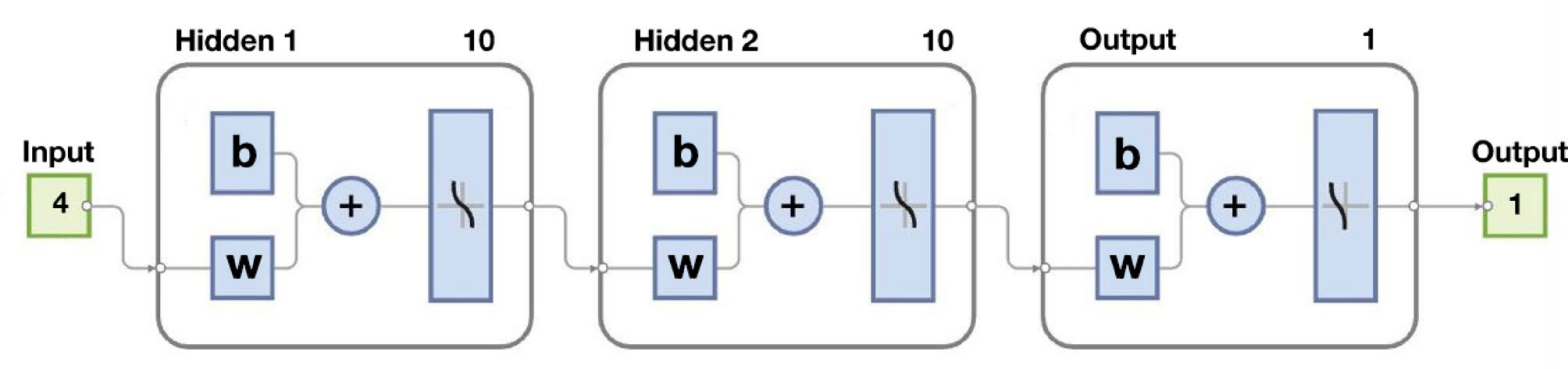
Proposed ANN architecture.

With 10 neurons in each layer, the network has the ability to effectively capture the features of the data. In Fig. 10, the two hidden layers and the corresponding number of neurons can be observed.

### Network training

In this proposed structure, the data is divided into two parts, 70% for training and 30% for testing. The feedforward neural network learns using the training data. This training is repeated several times until the prediction error is less than a certain number (0.1).

**Fig. 11:**
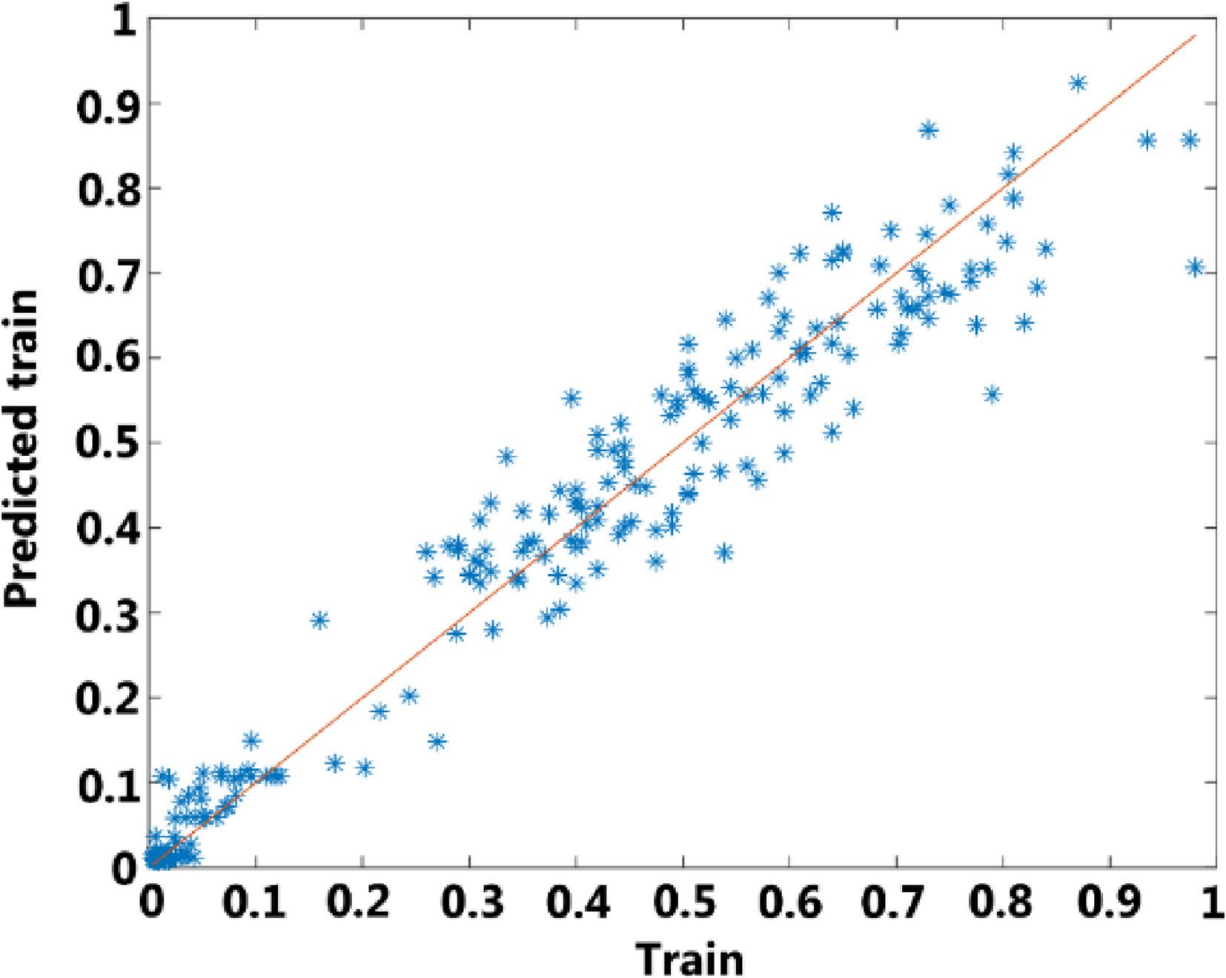
The prediction results of the proposed ANN model for train.

**Fig. 12:**
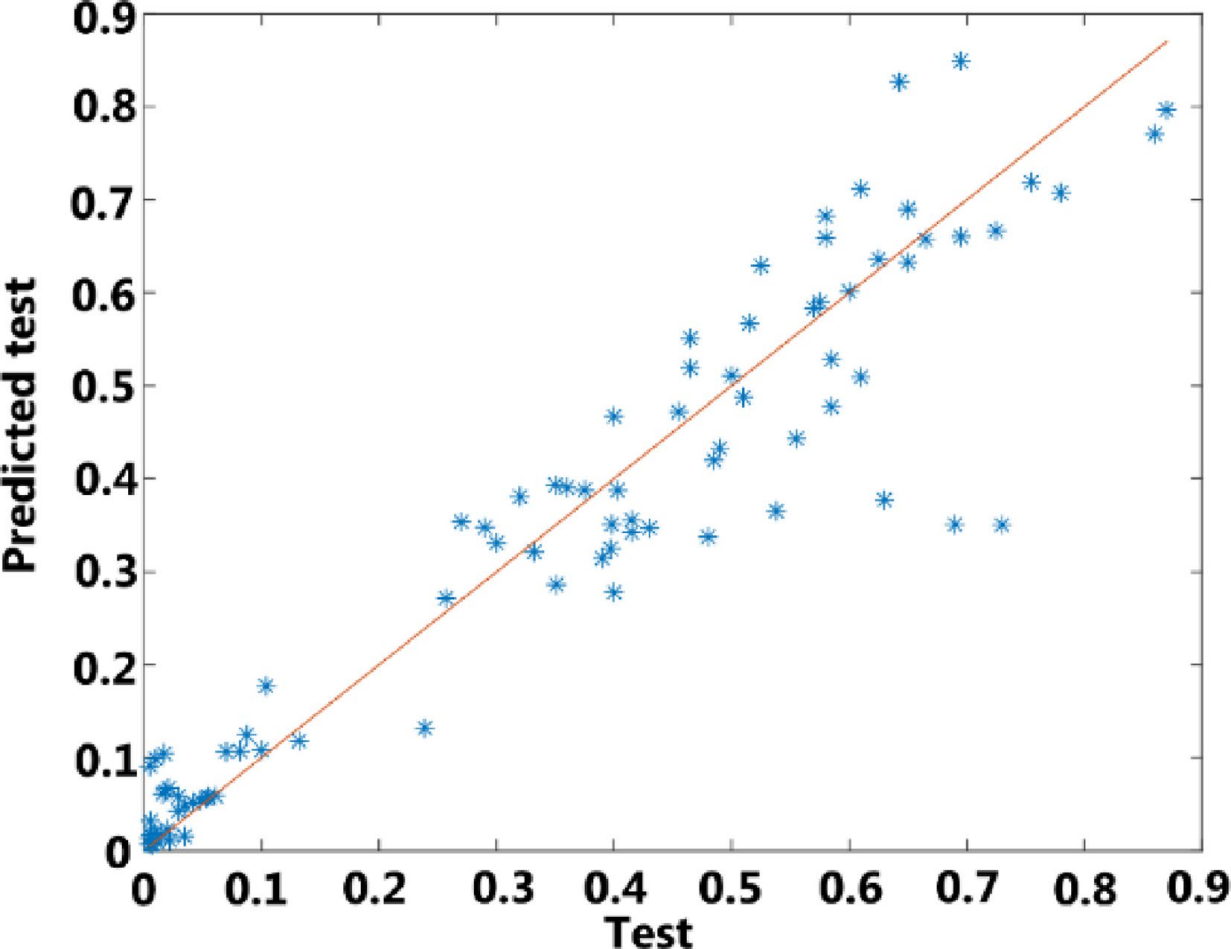
The prediction results of the proposed ANN model for test.

After training, the performance of the network is examined for both the training and testing parts (in Figs. 11 and 12). In these figures, the outputs predicted by the network are compared with the actual outputs. As can be seen, the predictions are in good agreement with the actual values, which indicates that the neural network has been able to model the XOR gate well.

## PSO-based XOR gate parameter optimization

The PSO algorithm is modeled after the collective behavior of birds and fish. In this method, several particles search together to find the solution. Each particle moves simultaneously with the others and adjusts its path in such a way that it uses both its own experience and the best experience of the entire group. The PSO algorithm does not require differentiation and is not limited to specific regions of the search space. These features make it very effective for optimizing complex and nonlinear functions and avoid getting stuck in local minima. The algorithm starts with random initializations for the position and velocity of the particles and then checks how well each particle is aligned with the optimization goal. Particles adjust their trajectories based on individual and swarm-wide best positions, gradually converging toward an optimal solution^42–45^.

In comparison to other optimization algorithms such as Genetic algorithm (GA) and Differential Evolution (DE), PSO is adopted to gate parameter optimization. GA operates on the basis of Darwinian natural selection and has advantages such as solving discrete and constraint-based problems, high population diversity, and also has a simple structure and implementation capability for a variety of problems^46^. However, it has disadvantages such as slow convergence for some parameters, and at the same time causes premature convergence in some specific conditions. In continuous problems, it usually performs weaker than DE or PSO^47,48^. Regarding DE, which is an algorithm based on the difference between population vectors to create new solutions, it has advantages such as high efficiency in continuous problems, fast convergence, simple structure and has disadvantages such as reduced diversity in multi-objective problems and unsuitability for discrete problems. However, PSO has fast initial convergence, simple and fewer parameter implementation, and has the ability to Hybridization^49^. Finally, this structure is very suitable for continuous problems with low to medium dimensions. In overall, PSO is a light and computationally efficient optimization method for nonlinear behavior structures PCs.

**Fig. 13:**
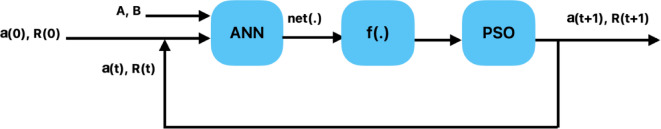
Parameter optimization framework: the integration of ANN and PSO.

The integration of PSO and ANN as a framework to compute the optimal values of the XOR gate (*a* and *r*) is depicted in Fig. 13. PSO is inherently an unconstrained optimization algorithm. However, here in this paper, *a* constrained version of PSO is developed so that the variables *a* and *r* are restricted within a predetermined range. As noted, PSO adopts an error or cost function to measure the suitability of each solution, represented by the pair (*a*, *r*). To align with our design objectives for the XOR gate, we employ PSO to minimize the error function presented as:

12 $$\:f\left(a,r\right)={\left(\text{n}\text{e}\text{t}\:\right(\text{a},\:\text{r},\:1,\:0)-1)}^{2}+{\left(\text{n}\text{e}\text{t}\:\right(\text{a},\:\text{r},\:0,\:1)-1)}^{2}+{\:\left(\text{n}\text{e}\text{t}\left(\text{a},\:\text{r},\:1,\:1\right)\right)}^{2}$$

where net (*a*,* r*,* A*,* B*) is the output of the trained ANN to design parameters *a* and *r*, and inputs *A* and *B*. Indeed, the error for a given pair of (*a*, *r*) is a combination of errors in three distinct states of the inputs.

**Fig. 14:**
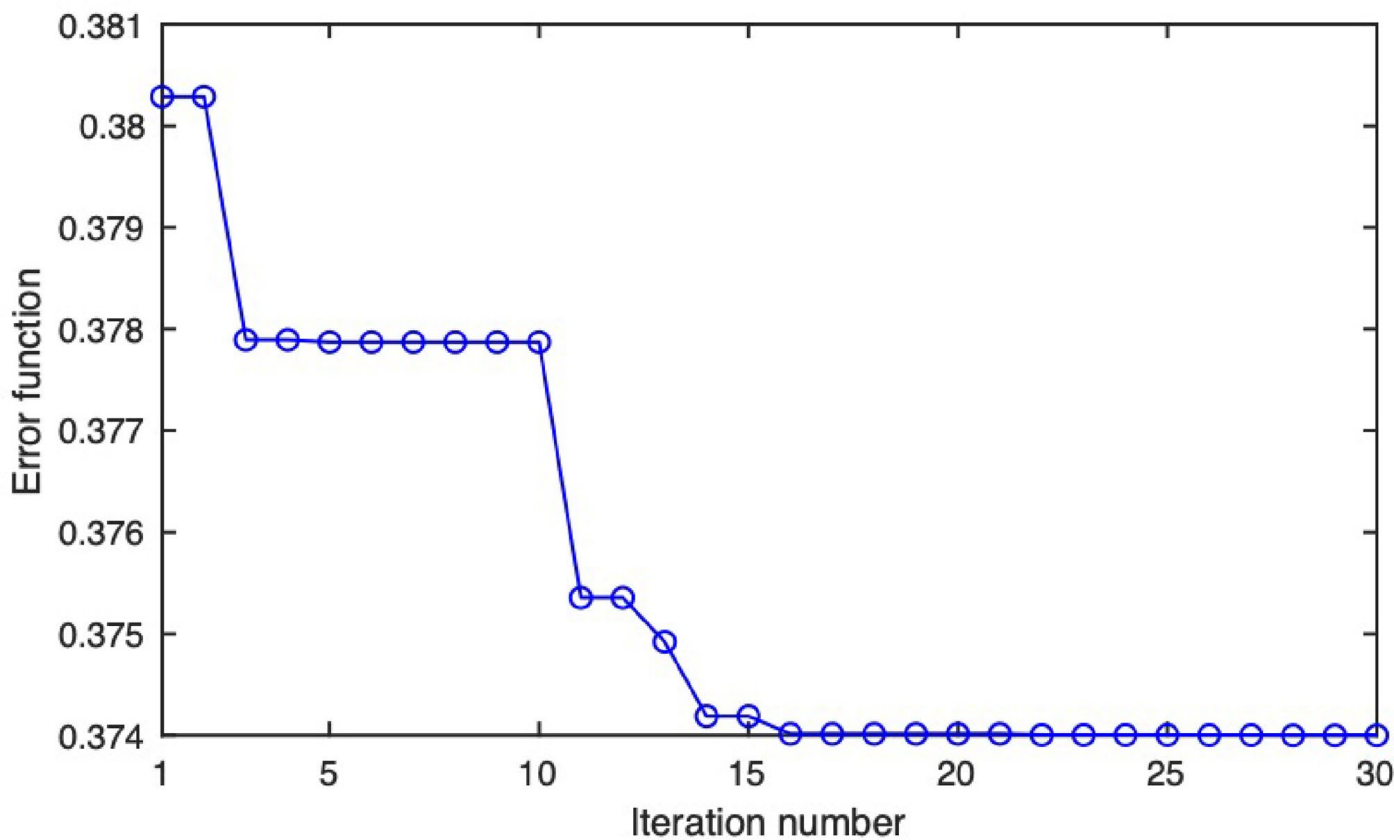
PSO convergence: error versus iteration.

The framework in Fig. 13 is initialized with a given pair of (*a* (0), *r* (0)) values. At each iteration, the trained ANN computes the output to these values in three states: (*A = 1*,* B = 0*), (*A = 0*,* B = 1*), and (*A = 1*,* B = 1*). These outputs are then used to derive the equivalent error function in Eq. 12. The PSO algorithm employs its particles and optimization techniques to find the next pair of (*a*, *r*) values. These values are then passed back to the ANN, and the error function is evaluated once more. The process continues until the error function converges. Figure 14 presents a typical convergence of the error function, where convergence occurs within less than 20 iterations.

## Results and discussion

The optimal design parameters (*a*, *r*) derived from the proposed optimization framework proposed in Fig. 13 are presented in Table 3. This table presents the optimal values of these parameters for five district percentage values of the training set. The outputs of the photonic crystal XOR gate with the derived designed parameters are also presented for three district states of the inputs. Considering a range of percentages of the training set as a hyperparameter in the ANN modeling enhances the model’s diversity and performance.

**Table 3:** Output of optimized parameters (ANN-PSO).

Training	a(µm)	*r*(µm)		Output	
			A = 1, B = 0	A = 0, B = 1	A = 1, B = 1
50%	0.5336	0.0999	0.734	0.870	0.007
60%	0.5329	0.0974	0.700	0.800	0.011
70%	0.5333	0.0952	0.940	0.995	0.017
80%	0.5317	0.0977	0.680	0.790	0.010
90%	0.5311	0.0990	0.630	0.740	0.009

Considering the results in Table 3, we notice that the most appropriate outputs are obtained when the training set comprises 70% of the dataset. In this value, the outputs for cases *A = 1*,* B = 0* and *A = 0*,* B = 1* are closest to one, while for *A = 1*,* B = 1*, the output is closest to zero. The optimal design parameters associated with these values are *a =* 0.5333 μm and *r* = 0.0952 μm.

The optimized structural values ​​obtained from the ANN-PSO framework – in particular *a* = 0.5333 μm and *r* = 0.0952 μm – are chosen based on numerical optimization without considering fabrication limitations. Indeed, in the PSO optimization technique, the search space for the optimization parameters is considered continuous. However, there is always some difference between simulation and fabrication results^50]– [51^. For physical implementation of the structure, the achieved dimensions must be rounded to the nearest values ​​compatible with the fabrication process (such as lithography). Although the optimized structural parameters using the ANN-PSO framework provide reasonable performance, for physical fabrication, it is essential that the optimized values ​​be rounded to the nearest manufacturable dimensions.

**Fig. 15:**
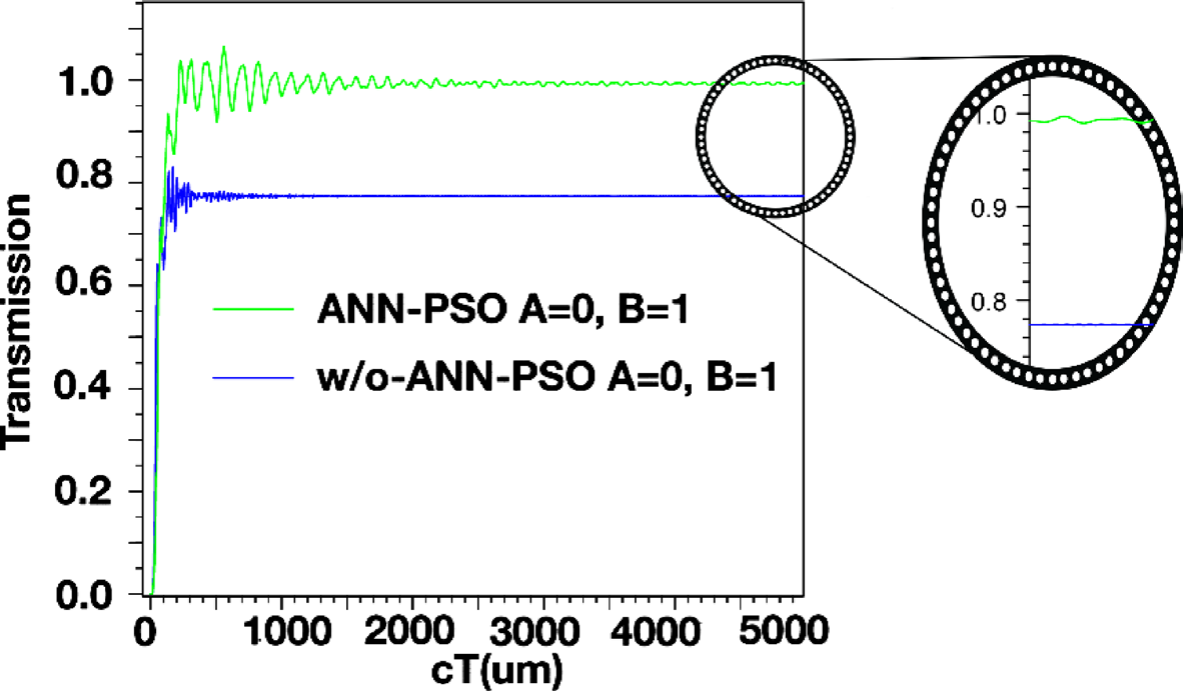
Output of XOR structure in *A = 0*,* B = 1* with optimal and non-optimal design parameters.

**Fig. 16:**
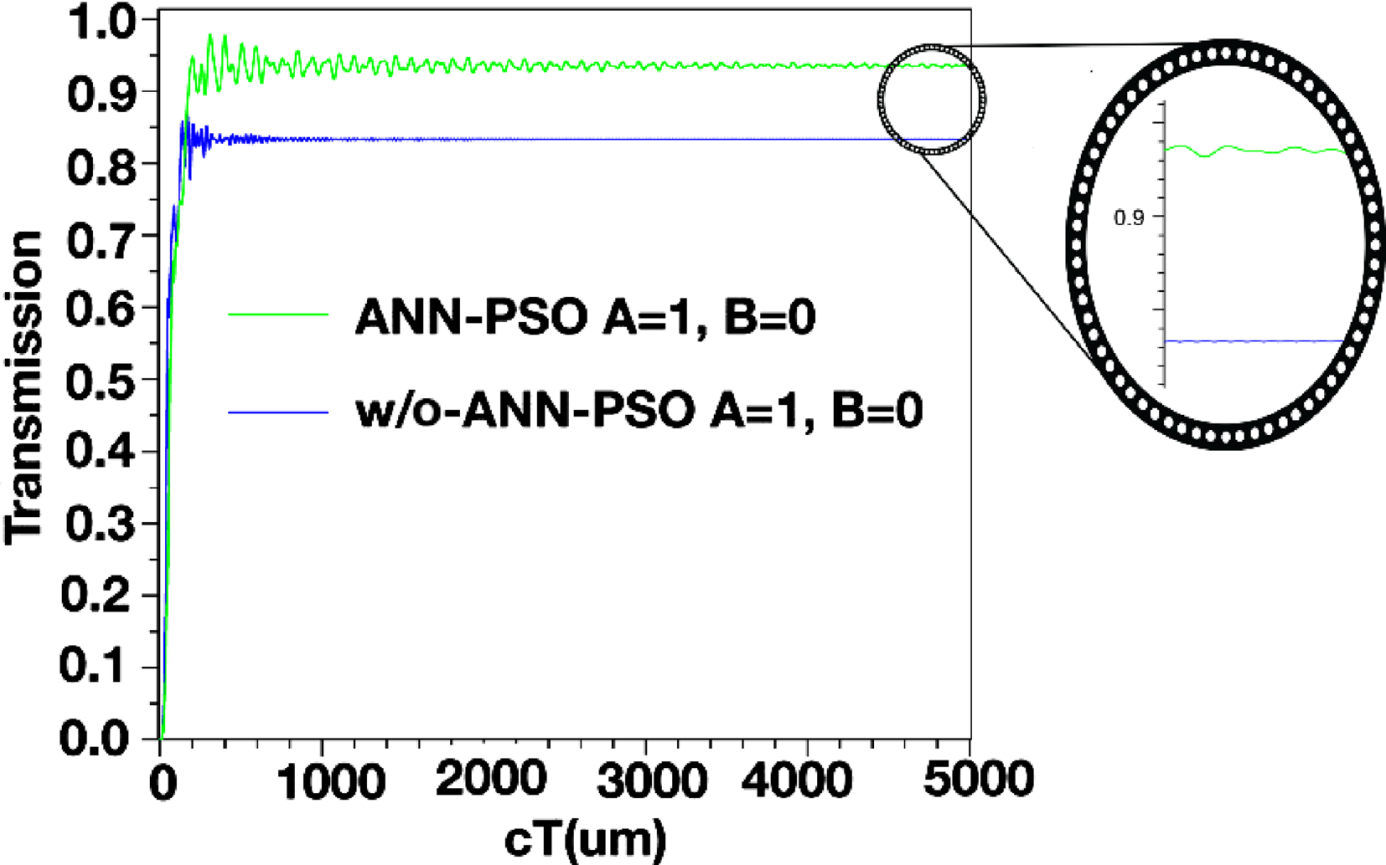
Output of XOR structure in *A = 1*,* B = 0* with optimal and non-optimal design parameters.

**Fig. 17:**
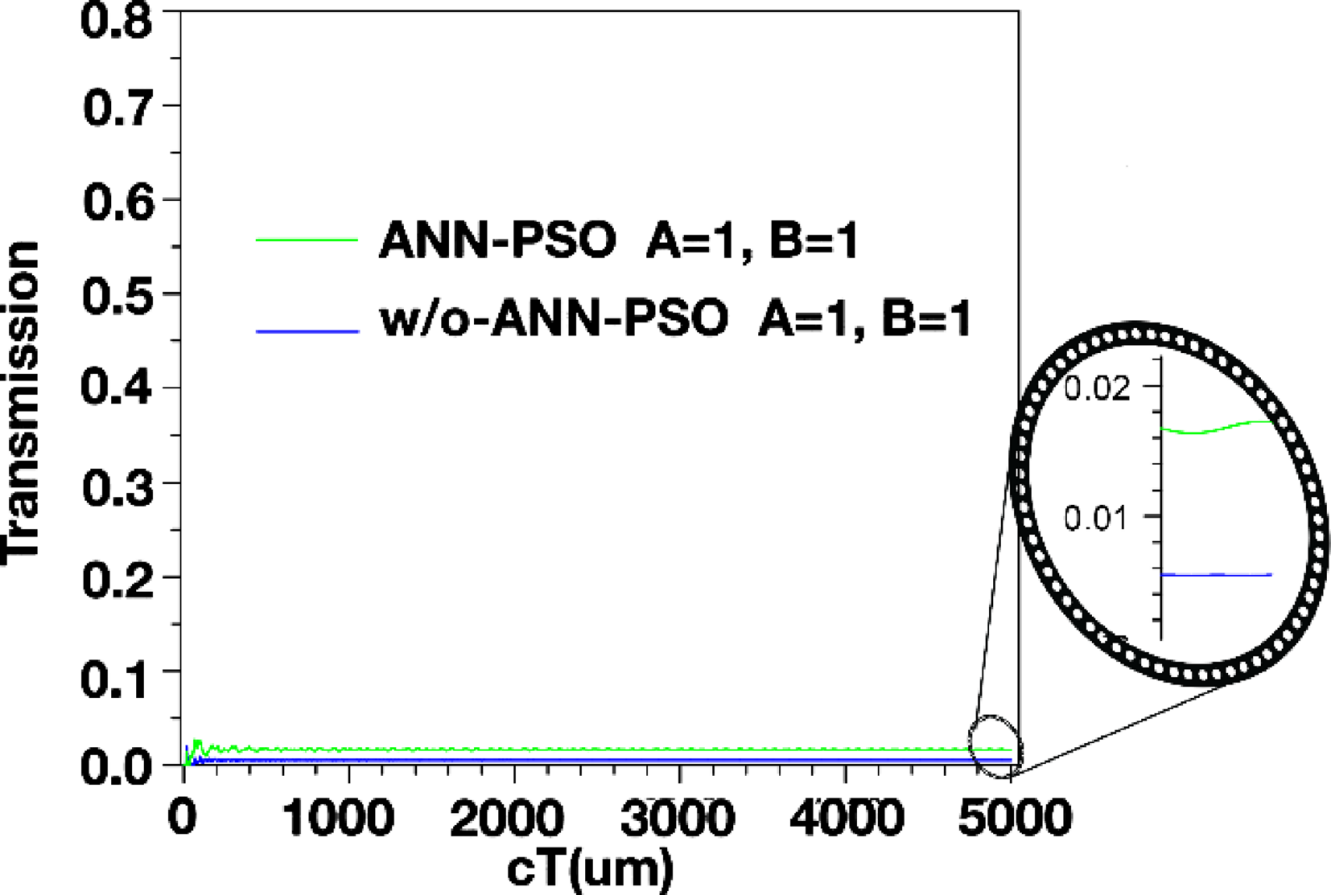
Output of XOR structure in *A = 1*,* B = 1* with optimal and non-optimal design parameters.

Figures 15 and 16, and 17 compare the outputs of the PC structure with optimal design parameters obtained from the proposed ANN-PSO to those obtained from simulations in Sect. 3. As observed, the gate outputs with optimal design parameters exhibit significant improvements.

Analyzing the structure’s output reveals a notable improvement after modeling it using ANN and subsequently optimizing it using the PSO algorithm. The optimized structure produces outputs that closely resemble the expected behavior of an XOR gate (as shown in Table 4): values near one when the output should be one and values very close to zero when the output should be zero.

**Table 4:** The final and optimal output of the desired structure.

A	B	Normalized Output	Logic value	CR (dB)
0	0	0	0	17.947
0	1	0.995	1	
1	0	0.935	1	
1	1	0.015	0	

Table 5 presents a comparison between the proposed XOR gate and other related works. The table evaluates four logical states to assess the performance of all-optical gates. The results show that the proposed gate, which utilizes the proposed ANN-PSO optimization framework in its design, outperforms the alternatives. The output values in our structure are closer to target values in comparison to other references.

**Table 5:** Review and comparison.

Ref	Technique	A = B = 0	A = 1, B = 0	A = 0, B = 1	A = B = 1	CR (dB)
^52^	PWE-FDTD	0	0.812	0.545	0.301	-
^53^	Finite element method (FEM)	0	0.298	0.299	0.00000202	49.5
^54^	Phase change	0.13	0.522	0.596	0.157	-
^55^	PWE-FDTD	0	0.962	0.952	0.002	26.77
^56^	-	0.30	0.436	0.431	0.210	11.56
^57^	PWE-FDTD	0	0.676	0.676	0.243	4.44
^58^	-	0	0.90	0.85	0.05	12.31
This work	PWE-FDTD	0	0.935	0.995	0.015	17.947

The maximum CR is 17.947 dB, which is lower compared to some structures and the reason for this low value of CR is that they use more complex structures, e.g^53^. , is based on valley photonic crystal (VPC) and uses a hexagonal lattice of holes in a silicon substrate, breaking the inversion symmetry to create a topological phenomenon and open a gap at the Dirac point. In^55^, a strong dependence on nonlinear properties is observed, as the structure is designed based on a nonlinear resonator whose operation depends on the Kerr effect. In contrast, our structure is intentionally simple, symmetric and avoids using complex elements such as loops or topological components.

The initial structure parameters derived from PWE-FDTD are input to the PSO optimization algorithm. The objective of this algorithm is to discover more optimal parameter combinations that enhance efficiency or optimize the band gap. While the initial design is based on PWE-FDTD, as presented in Table 5, the results obtained through optimization significantly improves the structure’s performance. The AI-driven optimization improves the entire structure performance and its outputs, leading to enhanced efficiency and performance.

Table 6 presents a comparative analysis between the proposed structure and other machine learning-based methods. As seen, the output values in our structure are closer to target values in comparison to machine learning based methods in^59] and [60^. Unlike the methods in^59^ or^60^, which predict the output by modeling the gate behavior, the proposed structure combines an ANN with a PSO algorithm to improves the modeling accuracy and increase the quality of the outputs by finding optimal values for gate design parameters. The design of the proposed structure using accurate FDTD simulation data and 555 data points, which provides a good logical performance for the XOR gate. These results show that the proposed structure is superior to previous methods in terms of simplicity, accuracy, and efficiency, and is considered an effective step in the development of all-optical circuits based on PCs.

**Table 6:** Comparison with machine-leased based methods.

Ref	Technique	A = B = 0	A = 1, B = 0	A = 0, B = 1	A = B = 1
^59^	RNN	0	0.820	0.813	1 × 10^− 7^
^60^	KNN	1.202 × 10^− 12^	0.882	0.882	1.202 × 10^− 12^
This work	ANN-PSO	0	0.935	0.995	0.015

## Conclusion

Design parameter optimization for enhancing the performance of all-optical XOR gates has been explored. This optimization can be computationally intensive and time-consuming when conventional simulation tools are employed to determine the output for specified design parameters. The findings presented in this paper demonstrate that utilizing the modeling capabilities of ANNs to model the behavior of a XOR gate, coupled with parameter optimization using PSO, leads to an effective design in terms of accuracy and complexity. The results indicate that this integrated approach offers a more efficient optimization strategy for PC-based structures. The findings show that the output of the gate in the logical 1 state reaches its maximum value, while in the logical 0 state, it approaches zero. The proposed all-optical XOR gate produces output powers of 0, 0.995, 0.935, and 0.015 for the corresponding logical states. Additionally, the CR achieved in this structure is 17.947 dB.

## Data Availability

The datasets used and/or analysed during the current study available from the corresponding author on reasonable request.
